# Health Impact Assessment in protected areas: a proposal for urban contexts in Brazil

**DOI:** 10.1590/0102-311XEN087223

**Published:** 2023-12-04

**Authors:** Ana Schramm, Sandra de Souza Hacon, Andre Reynaldo Santos Périssé

**Affiliations:** 1 Escola Nacional de Saúde Pública Sergio Arouca, Fundação Oswaldo Cruz, Rio de Janeiro, Brasil.

**Keywords:** Health Impact Assessment, Protected Areas, Urban Zone, Community Participation, Avaliação do Impacto na Saúde, Áreas Protegidas, Zona Urbana, Participação da Comunidade, Evaluación del Impacto en la Salud, Áreas Protegidas, Zona Urbana, Participación de la Comunidad

## Abstract

The use of Health Impact Assessment (HIA) in the establishment of an urban protected area can enhance the positive impacts and mitigate the negative impacts resulting from its implementation. Brazil hosts some of the most important biodiversity hotspots in the world and the HIA may benefit biodiversity and human health. These areas are commonly created without any preceding survey to assess their impacts on health. Protected areas located in urban zones are essential to maintain environmental balance and quality of life in cities. It promotes positive impacts on health, providing ecosystem services and salutogenic benefits. However, they can generate negative impacts such as the violation of human rights, property speculation, spread of vectorial diseases, and psychosocial stress. Based on the identification of the potential impacts of urban protected areas on health and best practices, this qualitative and exploratory study justifies the use of HIA in urban protected areas, especially in the Brazil, and indicates the main elements for the construction of a methodological approach to contribute to the Sustainable Development Goals and one of its alternatives, the *Buen Vivir* approach.

## Resumo

O uso da Avaliação de Impacto à Saúde (AIS) na criação de uma área protegida urbana pode potencializar os impactos positivos e mitigar os impactos negativos resultantes de sua implementação. O Brasil abriga alguns dos mais importantes *hotspots* de biodiversidade do mundo e a implementação da AIS pode beneficiar tanto estas áreas como a saúde humana. As áreas protegidas urbanas são comumente estabelecidas sem qualquer avaliação prévia de seus impactos na saúde e são essenciais para manter o equilíbrio ambiental e a qualidade de vida nas cidades. Além disso, as áreas protegidas impactam positivamente a saúde, fornecendo serviços ecossistêmicos e benefícios salutogênicos. Contudo, podem gerar impactos negativos, como violação de direitos humanos, especulação imobiliária, disseminação de doenças vetoriais e estresse psicossocial. Com base na identificação dos impactos potenciais das áreas protegidas urbanas na saúde e nas melhores práticas para aplicá-las, este estudo qualitativo e exploratório justifica o uso da AIS em áreas protegidas urbanas, especialmente no Brasil, e indica os principais elementos para a construção de uma abordagem metodológica que contribua com os Objetivos de Desenvolvimento Sustentável e uma de suas alternativas, a abordagem *Buen Vivir*.

## Resumen

Usar la Evaluación del Impacto en la Salud (EIS) para crear un área protegida urbana puede potenciar los impactos positivos y mitigar los impactos negativos resultantes de su implementación. En Brasil se pueden encontrar algunos de los *hotspots* de biodiversidad más importantes del mundo e implementar la EIS puede beneficiar tanto estas áreas como la salud humana. Las áreas protegidas urbanas, en general, se establecen sin cualquier evaluación previa de sus impactos en la salud y son esenciales para mantener el equilibrio ambiental y la calidad de vida en las ciudades. Además, las áreas protegidas tienen un impacto positivo en la salud, proporcionando servicios ecosistémicos y beneficios salutogénicos. Sin embargo, pueden generar impactos negativos, como la violación de los derechos humanos, la especulación inmobiliaria, la propagación de enfermedades vectoriales y el estrés psicosocial. Con base en la identificación de los posibles impactos de las áreas protegidas urbanas en la salud y en las mejores prácticas para aplicarlas, este estudio cualitativo y exploratorio justifica el uso de la EIS en áreas protegidas urbanas, sobre todo en Brasil, e indica los elementos principales para construir un enfoque metodológico que contribuya a los Objetivos de Desarrollo Sostenible y una de sus alternativas, el enfoque Buen Vivir.

## Introduction

Protected areas are the main tool for in situ biodiversity conservation policies and for the preservation of cultures, territories, and traditional populations ^1^ ^,^ ^2^ ^,^ ^3^. Biodiversity, a key environmental determinant of human health, can provide health protection against the spread of infectious diseases, as well as offer a better quality of life for the areas under their influence ^4^ ^,^ ^5^ ^,^ ^6^. Protected areas are essential since their restricted exploitation conserves biological systems, maintaining ecosystem services and options for future sustainability that might otherwise be depleted, degraded, or destroyed ^7^. These areas are also the subject of various economic development policies ^8^ ^,^ ^9^. However, their establishment can threaten rights and livelihoods of people, allowing access for some but excluding others, generally the poorest ^10^ ^,^ ^11^. In this sense, there are many controversies about these areas since they can have positive or negative impacts on human health, depending on how they are implemented ^12^ ^,^ ^13^ ^,^ ^14^ ^,^ ^15^.

Although the role of protected areas in supporting human health is well understood ^16^, few policy implementation tools effectively use it to inform development decisions for protected areas aimed at ensuring human health and biodiversity conservation that is compatible with socioeconomic development ^9^. Impact assessment tools, such as the Health Impact Assessment (HIA), support decision-makers in analyzing the positive and negative impacts of interventions, and their consequences for policies, programs, and services, in urban and rural areas ^17^.

However, there are no specific guidelines or impact assessment tools that consider health in the establishment of protected areas. Even with the increasing use of other instruments, such as the Environmental Impact Assessment (EIA) and the Strategic Environmental Assessment (SEA), these tools only focus on issues such as public exploitation of natural resources; not however, addressing the full range of social determinants of health ^18^. They may include, at best, the dimensions of quality of life and well-being. In this sense, we defend using the HIA for the establishment of protected areas since these areas are commonly created and managed without any type of study that comprehensively assesses the impacts on health and socio-biodiversity ^19^.

The use of a methodological approach that emphasizes human health impacts for the areas of influence of a protected area is urgent. Biodiversity loss and wildlife markets increase the risk of disease spillover from wildlife to human populations, and the emergence of many of the new scourges of our times, such as HIV, Ebola, Nipah, SARS, H5N, and COVID-19, can be attributed to increased human impacts on nature ^20^ ^,^ ^21^. These issues are also strongly influenced by the climate crisis ^22^, which is a major driver of emerging and reemerging infectious diseases ^23^.

These impacts may be even greater in megadiverse countries such as Brazil, especially in cities and their limit, where urban sprawl leads to biodiversity loss by habitat fragmentation, while socioeconomic inequality increases. Studies in several countries show that 50% or more of the regional or even national biological community is found in cities, despite the intense transformation of the natural environment ^24^.

Brazil, a large-sized country, is at the top of the 18 megadiverse countries, with about 15 to 20% of the world’s biodiversity. It presents six terrestrial biomes with their respective ecosystems, namely the Amazon, Caatinga, Cerrado, Atlantic Forest, Pampas, and Pantanal, three large marine ecosystems ^25^, and a great sociocultural diversity, expressed in several ethnic groups and indigenous peoples, *quilombola* communities, riverines, and traditional agricultural producers, present in urban and rural areas ^26^. The Atlantic Forest and the Cerrado are global biodiversity hotspots ^25^. These biomes are located in the regions with the largest urban populations, in the Southeastern Brazil, where the Atlantic Forest predominates, and with the highest growth rate of urbanized areas (in the case of Cerrado) ^27^ ^,^ ^28^.

In Brazil, it is estimated that more than 61% of the population is concentrated in urban areas ^29^. The country’s rapid and unplanned urbanization has led to the emergence of informal settlements inside and around cities. Such settlements occupy riverbanks, hillsides, and wasteland, often with industrial environmental liabilities and fragile soils ^30^ ^,^ ^31^. Most of Brazilian urban agglomerations are located within or on the outskirts of protected areas, with poor sanitation and infrastructure, high levels of air pollution, lack of urban planning, and poor mobility. Moreover, violence and traffic accidents have led to a decreased quality of life and biodiversity loss ^7^ ^,^ ^32^. Populations living in these areas face a triple burden of disease, which further increases health inequities ^33^ ^,^ ^34^.

As the population of Brazil and the world becomes more concentrated in urban areas ^35^, human activities, such as consumer demand for food, water, and other natural resources, will also become more concentrated in these places. Global and local environmental changes, including climate change and biodiversity loss due to urbanization, and pressures on the natural environment, such as increased energy consumption and greenhouse gas emissions, deforestation, land degradation, and severe water stress, have multiple impacts on human health.

Given this scenario and the increase in social inequality in countries such as Brazil, it is important to focus on how to make cities more resilient, integrating research on poverty, food and water security, and ecosystem services. The HIA is the appropriate tool for intersectoral and multidisciplinary action, linking issues of climate change, air quality, and health risks and impacts to urban planning and management. Therefore, the use of the HIA in the establishment of protected areas in Brazilian urban and periurban areas can contribute both to mitigating and adapting to these local and global environmental changes, as well as to social inclusion and sustainable development, in the search for *Buen Vivir* [good living] goals and the achievement of international agreements such as the Sustainable Development Goals (SDGs) ^36^.

## HIA for protect areas in Brazil

HIA is a practical approach used to assess the potential health effects of a policy, program, or project on a population. Recommendations are made to decision-makers and stakeholders, to maximize positive health effects and minimize negative health effects of proposals, and their application in different economic sectors by using quantitative, qualitative, and participatory techniques ^37^. Studies show that the distribution of HIA is unevenly distributed worldwide due to contextual differences and forms of application ^38^ ^,^ ^39^ ^,^ ^40^. It is already well established as an autonomous process in some developed countries but is still poorly recognized and practiced in most low- and middle-income countries such as Brazil ^41^.

Winkler et al. ^42^ have found an upward trend in the use of HIA worldwide, with a several types of HIA and applications in different fields. However, the barriers to using HIA remain the same as those reported in previous studies: limited technical experience for practice; insufficient knowledge of HIA among decision-makers and public healthcare professionals; lack of HIA or health policies and regulations in other types of impact assessment. There is a clear understanding of the need to invest in capacity building for HIA, particularly in low- and middle-income countries ^38^ ^,^ ^41^. The authors point to the fundamental role of the World Health Organization (WHO) and the International Association of Impact Assessment (IAIA) in guiding the dissemination of the methodology, identifying good practices and the need to train the global network of impact assessment professionals ^42^.

Protected areas have not yet been the subject to HIA, but we found some HIA experiences in urban parks and green areas in Europe, Canada, and the United States ^43^ ^,^ ^44^ ^,^ ^45^ ^,^ ^46^ ^,^ ^47^. Nevertheless, other assessment tools and environmental studies have been used in protected areas implementation, such as the EIA, SEA, Social Impact Assessment (SIA), and Millennium Ecosystem Assessment (MEA) ^48^ ^,^ ^49^. Studies have qualitatively assessed the impacts of protected areas on the well-being and quality of life of populations, but there are few prospective and quantitative studies assessing these impacts ^9^ ^,^ ^18^ ^,^ ^50^. SIA has been used in protected areas implementation, particularly where traditional communities are involved ^51^.

Although HIA is not mandatory in Brazil, the Brazilian government published an HIA methodology guide for the environmental licensing process of large projects in 2014 ^52^, based on a joint effort between the Brazilian Ministry of Health and the Brazilian Ministry of Environment and Climate Change. However, HIA has only been developed in research institutions, where technical and scientific debates on how to make the tool applicable in Brazil are held. Some authors argue that it should be integrated into the EIA process, while others argue that HIA should be an autonomous process ^53^ ^,^ ^54^. Although the health component is explicit in the EIA, as an element of the socioeconomic dimension, and in urban management instruments, studies show that, despite the conceptual presence of health in these instruments, few elements and tools for its implementation can be found ^54^ ^,^ ^55^ ^,^ ^56^.

Brazil has little experience with HIA, mainly for environmental liabilities of large capital projects ^41^ ^,^ ^55^. Recently, HIAs on air pollution and other rapid HIAs in the urban context have been published ^56^. In Brazil, there are examples of SEA and SIA in federal protected areas ^57^. Jones et al. ^19^ recommend the use of this tool in the implementation of protected areas, as the creation of a new structure for the management and regulation of natural resources generates conflicts and imposes social impacts on local communities and other users. In the Brazilian environmental licensing process, the protected areas can be the subject of an EIA if they directly affect their area or can become beneficiaries of environmental compensation funds. This is also the case for projects financed by the International Finance Corporation (IFC), which uses HIA as the structuring centerline for its *Performance Standards in Social and Environmental Sustainability*. Performance Standard 6 provides guidelines for biodiversity conservation, considering the ecosystem services approach and adaptive management of mitigation measures ^58^. Similarly, in the process of land regularization, the Brazilian Forest Code ^59^ requires the definition of legal reserve areas and permanent conservation units, also required in urban management, by the *Neighborhood Impact Study*, demanded by the Brazilian City Statute ^60^.

Ultimately, if the assessment aims to mitigate human health impacts, either directly by the enterprise/policy or indirectly by the loss of ecosystem services, EIAs should be reformulated to consider health with the various social and economic dimensions ^18^. HIA is a model that allows for the integration of health, human well-being, and social determinants in their interrelationships with other dimensions of object analysis. Due to its principle of equity and, therefore, its distribution of impacts among vulnerable groups regarding gender, age, ethnicity, and socioeconomic status ^61^, it requires close participation of the affected populations, as well as other social actors. In this sense, this impact assessment model should be more widely used in Brazil. The epidemiological and exposure studies offered by HIA are essential in the context of multiple epidemics, which is characteristic of Brazil. Therefore, it is closer to the objectives of sustainable development, mainly to assess the relationship between biodiversity and health in urban areas.

HIAs conducted in developed countries, although they include social participation as part of the assessment process ^38^ ^,^ ^62^, do not give as much emphasis to this issue as is necessary for peripheral countries, such as Brazil. These countries are characterized by social inequality and poverty, where several social determinants of health simultaneously affect vulnerable populations, requiring a deepening of social participation and equity ^38^ ^,^ ^61^ ^,^ ^63^.

## Potential impacts on human health on urban protected area

Protected areas located in urban and periurban areas are essential for maintaining the environmental balance, and quality of life in cities. They promote positive health impacts, ecosystem services, and salutogenic benefits, such as thermal regulation, control of microclimates, surface runoff, noise reduction, air quality, maintenance in water resources, modulation of infectious diseases. Moreover, protected areas allow the preservation of historical, social, and cultural values and assets, and creation of opportunities for education, sport and leisure, economic, employments, income, and ecotourism, which are crucial for long-term urban sustainability ^64^ ^,^ ^65^ ^,^ ^66^ ^,^ ^67^.

Strong evidence indicates positive associations between biodiversity and psychological and physical well-being ^5^ ^,^ ^13^ ^,^ ^16^ ^,^ ^68^, as well as between ecosystem diversity and immune system regulation ^69^. In some places, physicians recommends to patients to spend some in natural areas ^70^. Protected areas have these beneficial effects and are potentially able to influence the formation of citizens by environmental education and health promotion actions, strengthening the political empowerment for the local management of public goods ^28^ ^,^ ^29^ ^,^ ^71^. Economic assessments of green spaces and protected areas in cities worldwide have found that nature “saves” billions of dollars in healthcare services ^66^ ^,^ ^72^, promotes ecotourism ^73^, and improves food security ^74^.

Conversely, if abandoned by public authorities, urban protected areas can negative affect health and the environment. As an example, we can cite deforestation and environmental degradation, which alters the hydrological and biochemical cycle of several micronutrients, along with air quality, which leads to thermal inversion and heat islands phenomena, increasing the risk of disease ^75^. Other negative impacts are related to violations of rights, land grabbing, real estate speculation, conflicts over land and water use, water and sanitation-related diseases, vector-borne diseases, psychosocial stress, and violence. This overlook on urban protected areas also contributes to the development of chronic noncommunicable diseases that overburden healthcare services and the economy ^11^ ^,^ ^16^ ^,^ ^64^.

These areas are under strong pressure from urbanization and exploitation of natural resources (mining, energy transmission networks, agribusiness, livestock farming), infrastructure works, and the conflicts between land use and livelihoods. At the same time that protected areas act as a harmonious space for recreation and quality of life, they can also be a source of environmental injustice and an instrument of alienation and exclusion of indigenous peoples, quilombolas, and rural communities, as well as migrants and other vulnerable groups in the cities ^10^ ^,^ ^76^ ^,^ ^77^. Disputes over claims to traditional territories, landless and homeless occupations, evictions, and the exclusion of protected areas, that can reduce poverty ^12^ ^,^ ^78^, or increase it ^79^.

Other conflicts concern the alliances of corporate capital, the consequent possibilities of “green grabbing”, which exacerbate the existing problems of land grabbing ^32^. There are innumerable processes of speculation and real estate valuation in urban and periurban protected areas, leading to gentrification, when the population living in or close to the area is displaced, that is, another form of social exclusion ^80^.

For all these reasons, it is crucial to recognize that health depends on the socioeconomic context, which will determine how biodiversity conservation is conducted. Protected areas can have different restrictive uses, ranging from the complete exclusion of human activities to the sustainable exploitation of natural resources. They also vary in shape, size, isolation, and type of management ^2^ ^,^ ^81^. All these characteristics affect both biodiversity and health impacts in different ways ^13^ ^,^ ^15^. For example, a study on protected areas in the Brazilian Amazon found that the incidence of malaria, acute respiratory infections, and diarrhea was significantly and negatively correlated to the area under strict environmental protection. On the other hand, sustainable-use protected areas may increase malaria since they increase exposure to mosquitoes ^82^.

The impacts of protected areas on health can be direct or indirect, local, or global, within or outside the areas. Most health impacts are expected at the periphery of the protected area, where a buffer zone is needed. A seminatural buffer zone has been advocated by Terraube et al. ^15^ to provide more co-benefits for both health and biodiversity. The emphasis placed on these buffer zones is even more important in urban areas since they regulate the impacts of land use, mitigate the effects of climate change ^83^, provide recreational and public spaces, and protect priority areas for biodiversity conservation.

**Figure 1:**
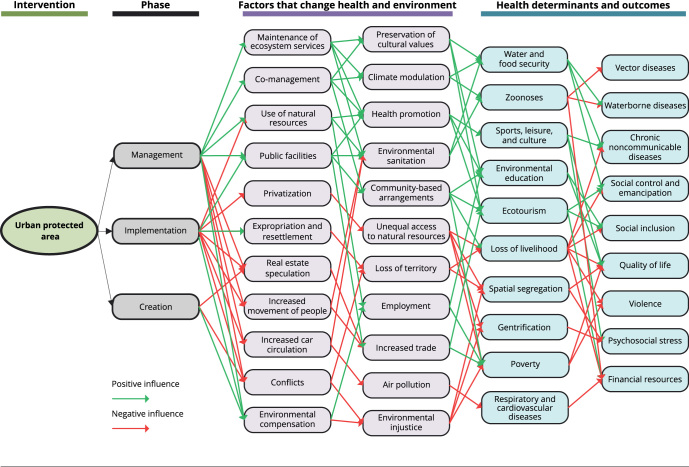
Diagram of potential impacts on health by an urban protected area.

Note: in the figure, impacts that can have a positive influence on the establishment of an urban protected area are shown in green, and impacts that can have a negative influence are shown in red. These impacts occur at different stages of the establishment of a protected area (creation, implementation, and management), which, in turn, have different factors that alter the environment, and these factors can lead to health outcomes and determinants.

In this review, we highlighted the challenge of identifying and characterizing health impacts associated with protected areas. Moreover, we considered the potential health impacts resulting from the establishment of generic urban and periurban protected areas, based on the HIA scope definition approach. This methodology defines the baseline basic health situation of the population groups that will be affected by the project, considering both health outcomes and socioeconomic determinants. It is based on data from literature, health systems, and dialogue with stakeholders ^17^. These data are organized and classified in a causal diagram (Figure 1), which helps to visualize how the different factors change the environment and affect health, the hypotheses to be investigated, and the multiple causes of an outcome. This analysis is useful to guide the analytical dimensions and activities of an HIA for protected area, and to identify the positive impacts that can be enhanced, and the negative impacts that can be avoided or minimized.

Figure 1 shows that impacts occur at different phases of the establishment of a protected area (creation, implementation, and management), which, in turn have different factors that change the environment, and these factors can lead to health outcomes and determinants. Each phase can impact on different directions and magnitudes, depending on how the process is conducted. Generally, impacts on the creation phase are related to the political arena. What impact will environmental studies have? How much money must be invested? Who will be able to participate in the process? The positive impacts are related to the activation of the network, an opportunity to initiate a shared management, socio-environmental and health diagnosis, knowledge of the territory, and allocation of environmental compensation resources. The negative impacts are linked to the exclusion of the population affected by the process, real estate speculation, and expectations of the proposal that create uncertainty.

Generally, the implementation phase can have the most negative impacts: increased resettlement, expropriation, spatial segregation, restricted access to and use of natural resources, and higher infrastructure costs. In the management phase, the impacts are likely to be more positive, considering an ideal scenario, with broad social participation, combined with local development projects that can have a positive impact on quality of life of the local population and the maintenance of ecosystem services. On the other hand, this phase may involve a loss of livelihoods and identity for local people. However, the nature and aspects of the impacts will depend on the way that the protected area is established and the involvement of the local population in determining the distribution of health risks and of access to natural resources. In this regard, local actors must include their demands in the process, to avoid territory loss, spatial segregation, gentrification, increasing social inequality, and health inequities.

## The main elements for the construction of HIA for urban protected areas in Brazil

It is estimated that the Brazilian protected areas, legally designated by the public authorities, occupy more than 37% of the national territory, considering the conservation units, indigenous lands, *quilombola* communities, and agrarian reform settlements. However, protected areas in Brazil present a scenario of poor effectiveness, being created for reasons other than conservation itself ^84^ ^,^ ^85^. In many cases, they are constructed to mitigate environmental liabilities for strictly political interests, generating the so-called “paper parks” ^86^ ^,^ ^87^. The lack of environmental studies to support the creation and management plan of protected areas, as well as the lack of participation of the local population, partly explain this scenario ^88^ ^,^ ^89^. Furthermore, this situation is aggravated in Brazil due to the inapplicability of laws and the relaxation of environmental and social policies.

The process of creating protected areas in Brazil implies prior environmental studies to characterize the situation of the physical, biological, and socioeconomic environment of the area, followed by the indication of the type and the polygonal proposal of the protected area. Therefore, when it is carried out, it involves an impact assessment, but only a simple diagnosis. An impact assessment analyzes (in terms of its nature, shape, duration, scope, cumulative and synergistic properties, magnitude, importance, and likelihood of occurrence), proposes mitigation and compensation measures, and monitoring programs. Box 1 shows a script to guide the elaboration of an HIA approach for the establishment of Brazilian urban protected areas. Some considerations on the elements that characterize it:

**Box 1:** A script to guide the elaboration of an Health Impact Assessment (HIA) approach for the establishment of Brazilian urban protected areas.

PHASES	DIMENSIONS	DESCRIPTION
Communication	Communication and governance	Continuous and specific communication and training strategies for each group of actors. Knowledge management (popular, technical, and scientific). Educational and scientific dissemination materials. Health promotion activities and participatory planning workshops.
Screening	Network of actors	Multidisciplinary and community-based HIA leadership management group. Network of local, governmental, private, and institutional actors. Mobilization and awareness-raising actions; initial agreement; pact; partnerships. Define evaluation scope and requirements.
Scope	Biota	Define evaluation scope and requirements.
	Physical environment	Diagnosis of water, air, and soil. Contamination of soil, water and air, erosion. Archaeological goods. Diseases (water, vector, noncommunicable diseases, cultural). Areas at risk.
	Climate	Atmospheric and climatological variables. Changes in the microclimate. Correlation with climate-sensitive diseases. Climatic risk areas. Feasibility of applying the IPCC recommendations.
	Sanitation	Diagnosis of basic and environmental, rural and urban sanitation, including local resources for the improvement and sustainable technologies. Situational and trend analysis related to health.
	People	Directly and indirectly affected populations. Interested stakeholders. Socioeconomic profile. Identification of local assets and resources (skills and competences). Social network analysis. Identification of macro- and micro-territorial scales (stratification in CAPs). Social cartography. Racism, violence, unhealthy environments, violation of rights, precarious work, unemployment, quality of life, and social cohesion.
	Diseases	Analysis of diseases: water, vectors, sexually transmitted, noncommunicable, mental health, alcohol and other drugs, COVID-19. Access to healthcare services. Household survey. Epidemiological profile and social determinants of health for the health baseline. Spatial distribution of diseases between groups. Perception of health risk.
	Urban infrastructure	Diagnosis of urban and rural infrastructure: housing, mobility, accessibility, access to services, security, employment, educational and leisure equipment, cultural and immaterial goods, neighborhood study. Characterize local assets (availability, distribution, and quality) and those to be maximized. Relate these assets to accidents/injuries, air pollution, noise pollution, psychosocial stress, climatic comfort.
	Policies, projects, and programs	Integrated analysis of PPPs. Urban, environmental, and health policies. Private and public real estate development. Public works and social interest. Sustainable development programs. Cultural and health facilities. Conflicts of interest in management. Local assets and resources. Opportunities for protected area and social inclusion. Public community partnerships.
	Access to natural resources	Analysis of the types of use, distribution, and access to available natural resources and related conflicts.
	Land use and occupation	Land analysis. Driving forces. Identify conflicts and social demands for land regularization. Possible processes of expropriation, resettlement, migration, urban expansion. Guidelines for inclusive zoning.
Risk analysis and mitigation	Ecosystem assessment	Assess needs and support capacity for water and food security, climate change, multiple endemic diseases. Valuation of ecosystem services. Health risk and impact assessment. Development of health and environment indicators (SDG, *Buen Vivir*).
Decision-making	Plans and recommendations	Proposal for protected area, polygon, and zoning category. Mitigation and adaptation plans (land, environment, and health). Protected area implementation plan. Mitigation and adaptation programs. Protected Area Management Committee. Terms of Reference for the Management Plan and Protected Area Master Plan.
Implementation and monitoring	Protected area creation and implementation	Creation of the protected area and the Steering Committee. Implementation of plans and projects. Management plan. Sustainable development projects. Ecotourism and community-based tourism. Health impacts monitoring. Community health management plan. Longitudinal studies.
Evaluation	Evaluation	Evaluate the impact of the HIA process, the participation of social actors, and the plans and projects.
Adaptive management	Updating of plans and projects	Review and adequacy of ongoing plans, projects, and programs. Monitoring. Permanent agenda for the control of plans for the actors involved, and social control.

CAP: communities affected by the project; IPCC: Intergovernmental Panel on Climate Change; PPP: policies, projects, and programs; SDG: Sustainable Development Goals.

Source: based on Winkler et al. ^17^, Brazilian Ministry of Health ^52^, International Finance Corporation ^58^, Millennium Ecosystem Assessment ^49^, and The Conservation Measures Partnership ^91^.

• The HIA of a protected area is, at the same time, an assessment of a policy, project, and program. The administrative act of creation alone will not cause direct health impacts, but this act will trigger projects and programs necessary for the establishment of the protected area, which will cause other impacts.

• The establishment of a protected area is a political intervention that regulates access to natural resources in the area. It is a distributive and regulatory policy, and therefore highly conflictual and often expensive. Therefore, HIA should consider the conflicts of interest between private rights and the social function of the property.

• Retrospective and prospective HIA requires understanding the current problems in the area, defining the health baseline of the community around the protected area, identifying trends in the main morbidities, anticipating scenarios, proposing monitoring and sustainability plans for the protected area, and conducting longitudinal studies.

• It must use the science of conservation biology, the adaptive management approach, and ecosystem assessment, focusing on human well-being as the goal of conservation. Uncertain scenarios of global change, multiple epidemics, and social inequality must also be considered.

• The issues of the right to the city; housing and land; mobility; water, food, and nutrition security; air pollution; and climate change should be considered in relation to integrated health impacts in the context of multiple risks. It should be integrated with the watershed plans, sustainable development projects, agroecology, family farming, and traditional knowledge.

• It must be a tool for the potentially affected population to address the social determinants of health, providing evidence that leads to social inclusion programs, land regularization, employment, and income, in a *Buen Vivir* perspective.

• Obtain primary, qualitative, and quantitative data, which is essential in the current scenario of uncertainty and information overload that hinders access to reliable data. Active methodologies for the collection of qualitative data by gathering the voice of the affected populations ^90^.

• Communication as a transversal axis for HIA. Develop different strategies for each group of social actors and produce informative materials to broaden the social engagement and reach of the evaluation.

• Identify and strengthen the local experience, skills, and competences of local actors, especially those who are living inside and on margins of protected areas for their management. Actors must be involved in the entire assessment process, from the drafting of the terms of reference, and must have deliberative power.

This approach is considered action research since it supports the solution of local problems, while it is increasing the knowledge of the actors involved and producing science. The assessment should use a set of mixed methods, such as qualitative (interviews, participatory planning workshops, and social cartography) and quantitative methods (a cross-sectional study or home-based survey and an ecological study, that is, a correlation between environmental and health variables), as well as tools that recognize uncertainties and assess resilience ^91^. The whole process must be led with network of local actors and support of local volunteers, based on the principle of citizen science and institutional partnerships. The profile of the recommended technical team is composed of the local community, high school students, community health agents, managers, institutions, epidemiologists, sanitary, ecologists, social scientists, and communication professionals.

**Figure 2:**
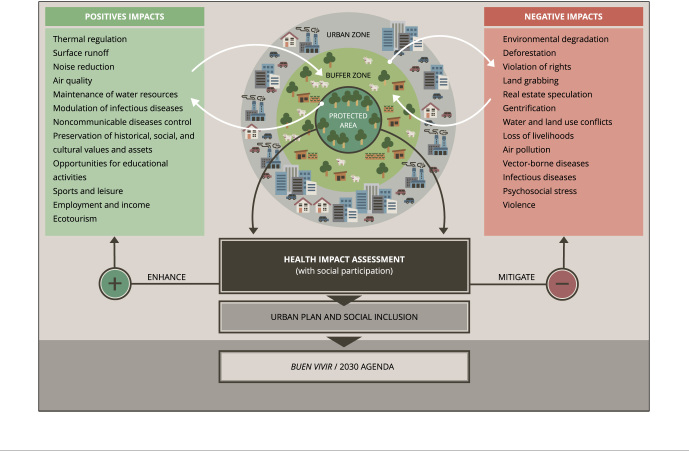
Graphical abstract of the Health Impact Assessment proposal for the establishment of an urban protected area.

Figure 2 provides a graphical summary of the proposal presented in this study, namely the need to use HIA in the establishment of an urban protected area, to enhance the positive impacts and mitigate the negative impacts arising from its implementation. In the figure, human health impacts occur in the border between urbanized and biodiversity conservation units. Emphasis should be placed on monitoring the response of biodiversity to human disturbance within protected areas, as well as on the periphery of protected areas and buffer zones, and on understanding how this, in turn, affects different dimensions of human health in different types of protected areas, according to the specificities of regional biodiversity ^92^. We highlight the importance of social participation of stakeholders and affected people in urban planning instruments. Following our model can contribute to the achievement of the SDGs and one of its alternatives, the *Buen Vivir* approach ^93^.

## Conclusions

This study aimed to justify the use of HIA in urban protected areas, especially in the Brazilian context. The synthesis of the literature on the subject helped to identify significant elements to support an HIA approach to urban protected areas, allowing to improve the processes of establishment of these areas to make the conservation of biodiversity compatible with human health and well-being. The potential for using HIA in urban protected areas is evident but remains to be explored to help address the most pressing global issues of climate, health, social, and environmental crises. The COVID-19 pandemic provides an opportunity to reaffirm the role of protected areas in reducing the risk of further zoonoses and supporting human health ^87^, and to establish protected areas in a context of urban expansion, which requires studies leading to urban planning integrated on biodiversity management and implementation of surveillance systems for early detection of emerging infectious disease events. This may also be an opportunity for the health sector to act in a different direction, triggering the self-organization of vulnerable urban populations to resist the loss of rights and health inequalities. The main limitations of this study are the lack of studies that provide data on biodiversity, health monitoring in remote, periurban, and urban areas, in addition to case studies of HIA for protected area. It is also limited by ideological bias, but justified by the context of social inequality, and needs to be validated with local actors and experts. Finally, it is necessary to institutionalize HIA in Brazil.
